# Multisensory Connections of Novel Linguistic Stimuli in Japanese as a Native Language and Referential Tastes

**DOI:** 10.3390/ejihpe11030074

**Published:** 2021-09-02

**Authors:** Yan Yan, Yutao Yang, Misa Ando, Xinyi Liu, Toshimune Kambara

**Affiliations:** 1 Department of Psychology, Graduate School of Education, Hiroshima University, 1-1-1 Kagamiyama, Hiroshima 7398524, Japan; m205403@hiroshima-u.ac.jp (Y.Y.); m206537@hiroshima-u.ac.jp (Y.Y.); ryu191618@gmail.com (X.L.); 2 Program in Psychology, School of Education, Hiroshima University, 1-1-1 Kagamiyama, Hiroshima 7398524, Japan; m214658@hiroshima-u.ac.jp

**Keywords:** associative learning, meaningless words, gustatory features, native language, language learning, embodied language, Japanese, dual coding theory

## Abstract

Previous findings have shown essential connections between linguistic and gustatory stimuli for people with autism or lexical gustatory synesthesia. We examined the associative learning of novel linguistic forms in Japanese as a native language and tastes (candies and chocolates) for healthy people. Healthy subjects performed four phases: (a) evaluation phase of gustatory features; (b) learning phases of novel linguistic form and gustatory stimulus pairs (G) or novel word forms (W); (c) recognition memory phases linked with G and W; and (d) free recall phase for G and W. In the recognition memory phases, the performance scores of W were higher than those of G, while there was no significant difference between response times of G and W. Additionally, no difference between recall performances in G and W was also shown. A subjective evaluation of gustatory features (sweetness) negatively correlated with the recall score for linguistic forms connected to the gustatory feature, whereas the accuracy rates of the recognition memory phase in G positively correlated with those of the free recall phase in G. Although learning of novel linguistic forms is more efficient than learning of the relationships between novel linguistic forms and tastes, gustatory features influence the free recall performances of linguistic forms linked with the tastes. These results may contribute to future applications to word learning not just for patients, but also healthy people.

## 1. Introduction

Most content words are composed of connections between linguistic forms (e.g., spoken, written, and other forms) and perceptual (e.g., visual, auditory, haptic, gustatory, and olfactory) and emotional features (e.g., [1,2]), although linguistic forms may not be directly linked with perceptually imageable features in function words (e.g., the). Associative learning of novel linguistic forms and perceptual features influences the recognition memory and recall performances of novel linguistic forms for healthy subjects [3,4,5,6,7,8,9,10,11,12,13,14,15,16,17,18]. For example, when subjects studied associative pairs of a color name (e.g., blue) and evaluative meanings (e.g., happy, beauty, and sweet), the color name could be linked with the evaluative meanings (e.g., positive evaluative meanings; see [3]). Another study showed that some factors (e.g., familiarity) could influence the learning of associations between words and meanings [15]. The research has mainly scrutinized learning of linguistic forms and pictorial, auditory, or haptic stimuli, whereas few studies have investigated learning of associations between linguistic forms and other sensory referents, including gustatory features.

Some studies report that gustatory referents effectively contribute to associative learning among multisensory features for people with autism or developmental disabilities (e.g., [19,20,21,22]), since the learning of linguistic and gustatory stimuli helps with their word learning. People with autism have language delay or impairment [23,24,25]. In a behavioral study, after three autistic children studied associative pairs of gustatory and visual features and associative pairs of visual and auditory features, two children could also study derived transitive pairs of gustatory and auditory features [21]. Another study reports that the Promoting the Emergence of Advanced Knowledge equivalent module, which is a development curriculum, may be effective while teaching adults with autism the relationships between multisensory features (gustatory, visual, and auditory stimuli) to improve their derived relational skills [22]. These findings about people with developmental disabilities are also consistent with a previous finding about healthy subjects [19].

In addition, previous studies of lexical gustatory synesthesia report that subjects who have word (lexical)-gustatory synesthesia can better remember associative pairs of linguistic forms and gustatory features compared with controls (e.g., [26]). Word (lexical)-gustatory synesthesia is a condition in which persons feel phantom tastes when reading, speaking, hearing, and imagining words (e.g., [26,27,28,29,30]). A neuroimaging study reported that the left anterior insula and left superior parietal lobule were activated during the word (lexical)-gustatory synesthesia experience [31]. These findings suggest that people with word (lexical)-gustatory synesthesia strongly link linguistic forms with gustatory features. However, it is still unclear whether the associative learning of novel linguistic forms and gustatory stimuli influences the recognition memory and free recall of the linguistic forms for healthy human subjects. Therefore, this research aimed to explore what the effects of associative learning of novel linguistic and gustatory features are in recognition memory and free recall phases.

In this experiment, we probed the effects of associative learning of novel linguistic forms and gustatory features on the recognition memory and free recall phases of linguistic forms. The behavioral experiment consisted of four phases: an evaluation phase of gustatory stimuli, a learning phase, a recognition memory phase, and a free recall phase. First, healthy Japanese subjects subjectively evaluated 20 gustatory stimuli on ten types of five-point semantic differential scales (sweetness, hardness, coolness, sourness, astringency, spiciness, familiarity, preference, arousal, and deliciousness, e.g., [32,33]). Second, the subjects studied associative pairs of novel linguistic forms and gustatory stimuli (G), and also studied novel linguistic forms only (W). Thirdly, at each recognition memory phase after the learning phase, the subjects evaluated whether each linguistic form appeared in the learning phase. Finally, at the free recall phase, the subjects wrote each linguistic form that they remembered. In this study, we could not collect additional samples due to the COVID-19 pandemic. This study may be recognized as the first preliminary and replicated experiment on associative learning of novel linguistic forms and gustatory stimuli for healthy subjects, since previous studies were conducted to study the learning of linguistic and gustatory stimuli for people with autism or developmental disabilities [19,20,21,22]. We had two predictions. First, when subjects studied the stimuli of G and W, subjects would remember and recall the linguistic forms of W more than the linguistic forms of G in a single day. This is because attentional loads to sources (gustatory stimuli) affect the performance of connections between items (linguistic forms) and sources (gustatory stimuli) greater than the performance of items (linguistic forms) only for healthy subjects (e.g., [16,17,34]). Second, we also predicted that the subjective evaluations of gustatory stimuli would affect the performance of the recognition memory and free recall of G. This prediction was congruent with previous findings. For example, our previous findings showed that preference of haptic stimuli negatively correlated with the free recall performances of linguistic forms connected to the haptic stimuli [17]. Other studies also suggested that emotional features affected the memory performances of associative learning [35,36].

In summary, we highlight the objective and hypotheses of the current research. The objective of the current psycholinguistic experiment was to test differences between recognition memory and recall performances of novel linguistic forms in G and W, and relationships between ratings of gustatory stimuli and performances in G. The first hypothesis was that the recognition memory and recall performances of W would be greater than those of G. The second hypothesis was that ratings of gustatory stimuli would be correlated with the memory performances in G.

## 2. Materials and Methods

### 2.1. Subjects in the Behavioral Experiment

Sixteen students (13 women; *M_age_* = 20.63; *SD_age_* = 2.03) took part in this experiment. All subjects were healthy and right-handed native Japanese speakers. All subjects provided informed consent before participating in the behavioral experiment. Each subject obtained a QUO card (gift card) of 500 Japanese YEN after the research. The research was approved by the Ethics Committee of the Graduate School of Education at Hiroshima University (code: 2019089).

### 2.2. Meaningless Words and Gustatory Stimuli

We prepared 80 meaningless (novel) words and 20 gustatory materials. The novel words were chosen from a study ([37]; see Supplemental Table S1). All the novel words consisted of two Japanese Katakana characters (e.g., nuyo). The meaningfulness of the chosen novel words ranged from 30 to 79 [37], while the non-association values in the chosen novel words ranged from 35 to 85 [37]. The gustatory stimuli (candies and chocolates) differed from each other (see Supplemental Table S2). Before the experiment, experimenters checked whether they could detect gustatory differences among stimuli for the control of gustatory stimuli. As shown in the Results section, the subjective evaluations of gustatory stimuli (e.g., familiarity) were also different among stimuli or among subjects. These gustatory stimuli were selected from supermarkets near Hiroshima University.

The novel linguistic forms were separated into four word-lists (word-list 1, 2, 3, and 4; see Supplemental Table S1) of 20 words each. Among the subjects, we counterbalanced the four word-lists as word-lists of G, W, and novel word conditions in the recognition memory phases of G and W (GN and WN). In a sample group, word-list 1, 2, 3, and 4 were the G, GN, W, and WN, respectively. In another sample group, word-list 1, 2, 3, and 4 were the WN, W, GN, and G, respectively. GN and WN were only presented in recognition memory phases, whereas G and W were presented in learning and recognition memory phases.

### 2.3. Tasks in This Study

The study was conducted in a within-subjects design. Superlab was used on a Windows OS laptop for the presentation of the word stimuli and recording of performances in the phases. The phases were ordered as an evaluation phase of gustatory stimuli in G, as learning and recognition memory phases of W, as learning and recognition memory phases of G, and as a free recall phase. The presentation order of the word stimuli was randomized in each learning or recognition memory phase of each condition across subjects. The order of gustatory stimuli in the evaluation and learning phases was unchanged to accurately provide the gustatory stimuli to subjects. In addition, we reversed the order of gustatory stimuli between subjects. Subjects drank water between gustatory stimuli if they wanted, with the exception of three of them. The learning and recognition memory phases of G or W were conducted as a pair. For example, after the learning phase of G, subjects performed the recognition memory phase of G. Subjects had a minute rest break after the learning phase of G and W. Throughout the study, the trials in G and W were not mixed.

**Figure 1:**
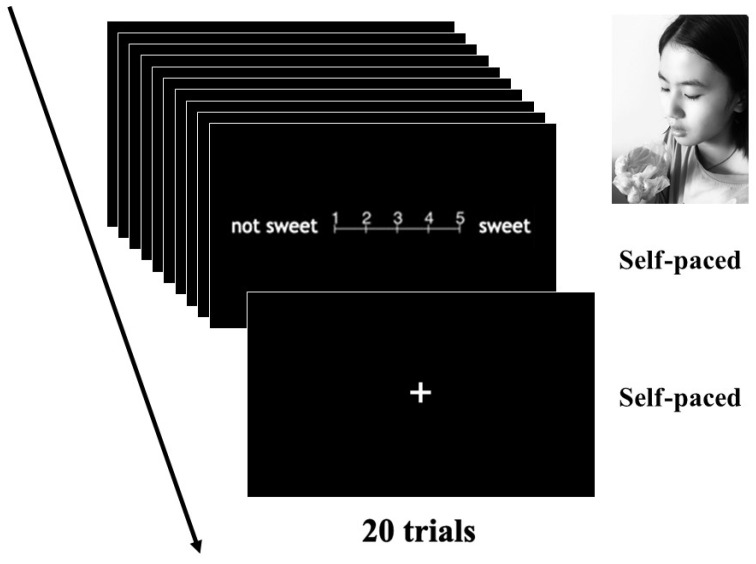
The flow of the evaluation phase.

In the evaluation phase, subjects made ten subjective evaluations to presented gustatory stimuli (candies and chocolates) using semantic differential scales (see Figure 1; [32]). Each semantic differential scale consisted of five points. First, an experimenter wearing clean latex gloves put each candy or chocolate directly onto each subject’s hand. Each subject also wore clean latex gloves. Second, each subject put the candy or chocolate in their mouth and tasted it. Third, each subject evaluated the candy or chocolate using each 5 point semantic differential scale. We based each semantic differential scale on other studies [32,33,38,39,40,41,42]. The 10 semantic differential scales linked with sweetness (1: not sweet; 5: sweet), hardness (1: soft; 5: hard), coolness (1: not cool; 5: cool), sourness (1: not sour; 5: sour), astringency (1: not astringent; 5: astringent), spiciness (1: not spicy; 5: spicy), familiarity (1: unfamiliar; 5: familiar), preference (1: not like; 5: like), arousal (1: calm; 5: excited), and deliciousness (1: not delicious; 5: delicious). After evaluating the candy or chocolate, the subjects put it in a trash can. During the evaluation phase, a fixation was presented between gustatory stimuli as a rest, to decrease fatigue. Subjects focused only on the fixation in the presentation. The fixation was not shown between semantic differential scales in each gustatory stimulus. The duration of each semantic differential scale and the fixation was determined by the subjects pushing a key. 

**Figure 2:**
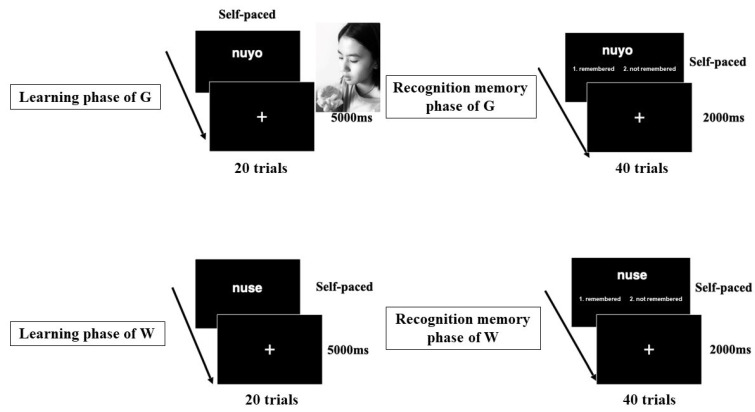
The flow of the learning and recognition memory phases.

After the evaluation phase of gustatory stimuli, the subjects performed the learning and recognition memory phases of G and W. In the learning phase of W, the subjects studied 20 novel linguistic forms. The stimulus duration was determined by the subjects pushing a key with their right index finger after studying each linguistic form. The key responses were recorded as the learning responses of words. Next, subjects performed the recognition memory phase of W. In the recognition memory phase of W, subjects judged whether the linguistic form was appeared in the learning phase by pressing 2 buttons (1: remembered; 2: not remembered). In the recognition memory phase of W, we used 20 novel words that did not appear in the learning phase of W (WN) and 20 words that were. During the learning phase of G, the subjects studied 20 associative pairs of a novel linguistic form and were offered gustatory stimulus (i.e., candies and chocolates; see Figure 2). During the learning phase of G, when an experimenter put a gustatory stimulus (a candy or chocolate) in each subject’s hand, the subject did not look at this gustatory stimulus. This is so that the visual features of the gustatory stimulus did not influence their learning of it. After the subjects put each gustatory stimulus in their mouth, they opened their eyes to memorize each associative pair between the stimulus and a novel linguistic form. The subjects memorized the novel linguistic form as a visually presented linguistic form and the gustatory stimulus (a candy or chocolate) as a gustatory presented referent (i.e., word meaning). The duration of stimuli was controlled by the subject pushing a key with their right index finger. Responses of keys were recorded as the learning responses of relationships between linguistic forms and gustatory stimuli. Next, subjects performed the recognition memory phase of G. In the recognition memory phase of G, subjects evaluated whether each word was present in the learning phase by pressing one of two buttons (1: remembered; 2: not remembered) that corresponded with a right index finger and a right middle finger. In the recognition memory phase of G, we used 20 novel words that were not present in the learning phase of G (GN) and 20 words that were. To decrease fatigue, a fixation was presented for 2000 ms between trials in recognition memory phases of G and W, while a fixation was shown for 5000 ms between trials in learning phases of G and W. During this time, subjects simply looked at the fixation. After the learning and recognition memory phases of G and W subjects embarked on the free recall phase, writing all the words that they remembered on an A4-sized paper. The subjects were explicitly required to write down only words that had been presented during the learning phase of G and W, as opposed to novel words presented in the recognition memory phase of G and W. We did not set a time limit for the free recall phase.

### 2.4. Data Analyses

Paired *t*-tests were conducted for accuracy rates and response times in G and W during the recognition memory and free recall phases. The design of this experiment was a within-subject design. All response times used in the analyses were response times of correct answers in the conditions. Paired *t*-tests were conducted on SPSS. As in previous studies (e.g., [43]), we also calculated correct response scores (CRS), which calculated hit rates (i.e., subjects correctly judged studied stimuli as studied stimuli) excluding false alarm rates (i.e., subjects incorrectly evaluated novel stimuli as studied stimuli). In addition, Cronbach’s alpha was calculated for clarifying the reliability of each scale. After the calculation of Cronbach’s alpha, correlation analyses were conducted to identify relationships between the ratings of each reliable scale and performance (proportion and response time) in the recognition memory and free recall phases. Since we set each proportion of each trial in the recognition memory and recall phases as 2 values (0: not remembered or not recalled; 1: remembered or recalled), point-biserial correlation analyses were conducted to investigate relationships between each rating of the reliable semantic differential scales and each proportion of each trial in the recognition memory and recall phases. The point-biserial correlation analyses were conducted as Pearson’s correlation analyses on SPSS software by using 2 values corresponding with each proportion of each trial in the recognition memory and recall phases. In addition, we performed Pearson’s correlation analyses between each evaluation of the reliable scales.

## 3. Results

### 3.1. Subjective Evaluations in Evaluation Phase

In order to clarify the reliability of each scale, the Cronbach’s alpha was calculated (Table 1). Cronbach’s alphas for sweetness, hardness, coolness, sourness, astringency, spiciness, familiarity, preference, arousal, and deliciousness were *α* = 0.90, *α* = 0.87, *α* = 0.87, *α* = 0.80, *α* = 0.84, *α* = 0.59, *α* = 0.89, *α* = 0.85, *α* = 0.90, and *α* = 0.88, respectively. Thus, we used 9 of these scales in correlation analyses, that is, sweetness, hardness, coolness, sourness, astringency, familiarity, preference, arousal, and deliciousness because Cronbach’s alpha for these scales was higher than 0.70 [44].

### 3.2. Proportions and Response Times of Hit Trials in Recognition Memory Phases

Regarding the accuracy rates of recognition memory phases, a paired *t*-test was conducted for comparing G with W (see Table 2). First, the recognition memory scores of W were higher than those of G (*t*(15) = 3.17, *p* < 0.01, *d* = 0.79). In addition, we compared the CRS of G with that of W (see Table 1). The CRS of W was also higher than that of G (*t*(15) = 2.95, *p* < 0.05, *d* = 0.74).

Regarding the response times of the recognition memory phases, we also conducted a paired *t*-test to compare G with W (see Table 1). We found that there was no significant difference between G and W (*t*(15) = 0.46, *p* = 0.65, *d* = 0.12).

### 3.3. Proportions in Free Recall Phase

The performances of G and W in the free recall phase were 0.07 (*SD* = 0.11) and 0.13 (*SD* = 0.14; Table 3), respectively. We found that there was no significant difference between the proportions of G and W (*t*(12) = 1.09, *p* = 0.30, *d* = 0.30).

### 3.4. Correlations

We found statistically significant correlations among evaluations of the gustatory stimuli (*p* < 0.05; see Supplemental Table S3). In addition, a subjective evaluation (sweetness) of gustatory stimuli was negatively correlated with the free recall score of linguistic forms connected to that stimuli (*p* < 0.05), whereas the proportions of the recognition memory phase positively correlated with the proportions of the free recall phase (*p* < 0.01). However, there was no significant correlation between the subjective evaluations of gustatory stimuli and the recognition memory performances of linguistic forms linked with the gustatory stimuli.

## 4. Discussion

We investigated the differences between the recognition memory and free recall performances of G and W and the effects of subjective evaluations of gustatory stimuli used in G. We found two main results. First, the recognition memory scores of W were greater than those of G. Second, a subjective evaluation (sweetness) of gustatory stimuli was negatively correlated with that of G in the free recall phase, whereas the accuracy rates of G in the recognition memory phase positively correlated with those of G in the free recall phase. In sum, the learning of novel linguistic forms may be better than the associative learning of novel linguistic forms and gustatory features. Furthermore, the recall performance of linguistic forms could also be affected by subjective evaluations of the gustatory referents connected to the linguistic forms. These findings support the two hypotheses outlined in the Introduction section.

### 4.1. Results of Recognition Memory and Free Recall Phases

In the recognition memory phases of this study, we found that the scores of W were higher than those of G, although there was no statistical difference between the response times of G and W in the recognition memory phases and between the proportions of G and W in the free recall phase. The results suggest that the learning of novel linguistic forms on its own is more effective than associative learning of novel linguistic forms and gustatory stimuli in a single day of learning. This finding supported the first hypothesis based on previous findings [16,17,34]. For example, a previous study suggested that the retrieval of source memory requires more attention than that of item memory (e.g., [34]). Our previous studies have also shown that memory performances of novel linguistic forms are higher than those of associative pairs of novel linguistic forms and referents including visual [16] and haptic stimuli [17]. Although the current and previous findings show that healthy subjects can learn novel linguistic forms on their own greater than with pairs of novel linguistic and referential features, people with sensory-related disabilities may show opposite or different results. For example, blind people can perform odor naming and non-visualizable paired word association tasks greater than healthy controls, since blind people may have superiority ability regarding nonvisual memories [45].

### 4.2. Correlations between Subjective Evaluations of Gustatory Features and Performance of Recognition Memory and Free Recall Phases

The current experiment showed that a subjective evaluation (sweetness) of gustatory features negatively correlated with the free recall performances of linguistic forms linked with the gustatory stimuli, this suggests that subjective evaluations of gustatory stimuli would affect the free recall of linguistic forms linked with that stimuli, and that evaluative responses to linguistic stimuli may be connected to sensory features of stimuli by associative learning. This finding also favored the second hypothesis based on previous findings [17,35,36]. A previous study showed that preference to haptic features negatively correlated with performance in the free recall phase [17]. Other studies have also shown that emotional features influence the memory performances of associative learning [35,36]. In addition, a previous study showed that evaluative responses of color names could be connected to those of words, including emotional valence (positive or negative emotions), by associative learning [3]. Thus, our findings suggest that a subjective evaluation of gustatory stimuli affects memory performances in the free recall phase after the associative learning of novel linguistic forms and gustatory stimuli.

People with sensory-related (e.g., gustatory) disabilities may find difficulty in multisensory learning of linguistic forms and referential (e.g., gustatory) features [46], since they cannot experience, store, and imagine perceptual features (e.g., tastes). For instance, Paivio and Okovita showed that blind people could not easily learn pairs of words with highly visual and low auditory imageries, compared with healthy controls [46]. On the other hand, people with sensory-related (e.g., gustatory) disabilities might have advantages for multisensory learning of linguistic and other sensory features (e.g., visual, auditory, haptic, and olfactory features). Paivio and Okovita reported that blind people could learn pairs of words with highly auditory imageries (e.g., music and gong) greater than healthy controls [46].

In addition, our findings reveal that the accuracy rates of G in the recognition memory phase positively correlated with those in the free recall phase. The results indicate that high performers of the recognition memory phase could also recall the linguistic forms in G better than low performers in the former phase. These findings are also consistent with a previous finding. An author and colleagues report that performances of associative conditions of words and pictures in recognition memory phases positively correlated with those of an associative condition of words and sounds in recognition memory phases [9]. Thus, high performers of associative learning of words and meanings could easily pass other phases related to memory performance, compared with low performers.

The current findings uniquely show the memory performances of linguistic forms on a single day of learning of linguistic and gustatory stimuli for healthy subjects, while previous studies show the memory performances of linguistic forms on a single day learning or multiple days learning of linguistic and visual, auditory, or haptic stimuli for healthy subjects [3,4,5,6,7,8,9,10,11,12,13,14,15,16,17,18]. Other research has also reported the learning effects of linguistic and gustatory features for people with word (lexical)-gustatory synesthesia [26] or with autism [21,22]. The current and previous results indicate that multisensory connections between linguistic and referentially perceptual stimuli (i.e., visual, auditory, haptic, olfactory, and gustatory) may be learned for both healthy subjects and patients. These results could provide behavioral evidence for a dual coding theory that linguistic forms (e.g., written and spoken forms) may be connected to referential (non-verbal) features (e.g., pictures, sounds, haptic feelings, smells, and tastes [1]). The multisensory connections between linguistic and referential features including sensorimotor and emotional features may play essential roles in the construction of primitive minds for human beings, if psycholinguists are able to explain that languages consist of links between linguistic (verbal) and referential (nonverbal) features as embodied languages. In future studies, psycholinguists need to conduct additional experiments for extending the dual coding theory.

On the other hand, we did not consider sound-symbolic (non-arbitrary) effects of the associative learning for novel linguistic forms and gustatory stimuli. Previous studies on sound symbolism have reported that specific verbal sounds or oral shapes that are required to produce certain verbal sounds are associated with certain perceptual and emotional evaluations [39,40,41,42,47,48,49,50]. A previous study found that specific verbal sounds increased expectations of certain tastes [50]. In addition, the sound symbolism of language may facilitate language learning [51,52,53]. If researchers in future experiments use novel linguistic forms non-arbitrarily connected to specifically gustatory images, the researchers may find that subjects easily learn connections between linguistic and specifically gustatory stimuli. Previous research also found relationships between typefaces of written phrases (e.g., rounder typefaces) and tastes (e.g., sweet; [54]). In this study, we did not focus on effects of typefaces in multisensory learning of linguistic forms and tastes, since we needed to only clarify the pure connections between linguistic forms and tastes as the first research step. However, if people apply typefaces already linked with specific tastes in the learning of linguistic and gustatory stimuli, the established connections between typefaces and tastes would influence the learning and test phases of linguistic forms and tastes. Furthermore, recent findings suggest that certain tastes (i.e., sweetness) may be linked with positive emotion words, while other tastes (i.e., bitterness, sourness, and spiciness) may be linked with negative emotion words [55].

### 4.3. Future Direction

Future studies may examine relationships between the associative learning and memory. Some studies suggest that verbal working memory associates with language comprehension (e.g., [56,57]) and learning of novel linguistic forms and pictures [18]. Since we could not collect additional samples after the COVID-19 pandemic, future studies may increase the sample size after the end of the COVID-19 pandemic.

## 5. Conclusions

We carried out a behavioral experiment to investigate the differences between associative learning of novel linguistic forms and gustatory features and learning of novel linguistic forms in recognition memory and free recall phases of the linguistic forms. In the recognition memory phase, the recognition memory scores and CRS of W were higher than those of G. Regarding the response times of the recognition memory phases, we found no significant difference between G and W. In addition, we found no significant difference between the accuracy rates of G and W in the free recall phase. On the other hand, a subjective evaluation of gustatory stimuli (sweetness) was negatively correlated with the recall score of linguistic forms connected to the gustatory stimuli. Finally, we also found that there was a positive correlation between accuracy rates of G in the recognition memory and free recall phases. In conclusion, the current findings disclose that the learning of novel linguistic forms on its own may be greater than the associative learning of novel linguistic forms and gustatory stimuli for a single day of learning with healthy subjects. Moreover, the recall performance of linguistic forms may be impacted by ratings of the gustatory stimuli connected to the linguistic forms. The findings of the multisensory connections between linguistic and referential connections may provide behavioral evidence to the dual coding theory, and other theories linked with embodied language and symbol grounding. In future studies, researchers may need to consider effects of verbal working memory and sample size on learning of novel linguistic and gustatory stimuli.

## Figures and Tables

**Table 1:** Descriptive statistics of 10 scales used in this experiment.

Scales (Item Means)	Alpha	*M*	MIN	MAX	Range	MAX/MIN	Variance	Items
Sweetness	0.90	3.64	2.25	4.88	2.63	2.17	0.48	20
Hardness	0.87	4.38	2.13	4.88	2.75	2.29	0.59	20
Coolness	0.87	2.67	1.31	4.06	2.75	3.10	0.88	20
Sourness	0.80	2.21	1.06	3.63	2.56	3.41	1.08	20
Astringency	0.84	1.72	1.13	2.75	1.63	2.44	0.26	18 *
Spiciness	0.59	1.31	1.06	1.75	0.69	1.65	0.05	9 *
Familiarity	0.89	3.25	1.31	4.88	3.56	3.71	1.15	20
Preference	0.85	3.43	1.50	5.31	3.81	3.54	1.08	20
Arousal	0.90	2.10	1.13	3.44	2.31	3.06	0.46	20
Deliciousness	0.88	3.44	1.31	4.69	3.38	3.57	1.00	20

*M*: mean scores; MIN: minimum value; MAX: maximum value. *: Items of astringency and spiciness were omitted for each calculation on SPSS, since the values of the omitted items were 1 which showed zero variance in the items. We calculated the values on SPSS.

**Table 2:** Results of the novel linguistic form and gustatory feature pairs (G), and novel linguistic forms (W) in recognition memory phases.

	G *M* (*SD*)	W *M* (*SD*)	*t*	*d*
Hit rate	0.64 (0.19)	0.75 (0.17)	3.17 *	0.79
False alarm rate	0.23 (0.16)	0.22 (0.14)	0.66	0.17
Corrected recognition score	0.41 (0.23)	0.57 (0.23)	2.95 *	0.74
Response time of hit trials	3542.87 (422.08)	3476.32 (578.95)	0.46	0.12

*M*: mean scores; *SD*: standard deviations; *t*: *t*-value; *d*: effect size; *: statistical significance. For corrected recognition scores, we computed the hit rate (i.e., subjects accurately evaluated each stimulus as those studied in G or W) minus the false alarm rate (i.e., subjects inaccurately evaluated each stimulus as those studied in GN or WN) for each subject. We performed the paired *t*-tests on SPSS.

**Table 3:** Results of the novel linguistic form and gustatory feature pairs (G), and novel linguistic forms (W) in the free recall phase.

G *M* (*SD*)	W *M* (*SD*)	*t*	*d*
0.07 (0.11)	0.13 (0.14)	1.09	0.30

*M*: mean scores; *SD*: standard deviations; *t*: *t*-value; *d*: effect size.

## Data Availability

The analyzed dataset is available on request to the corresponding author.

## Supplementary Materials

The following are available online at https://www.mdpi.com/article/10.3390/ejihpe11030074/s1, Supplemental Table S1: Japanese word stimuli (transliteration), Supplemental Table S2: Manufacturers and flavors of gustatory stimuli, Supplemental Table S3: Results of correlation analyses (*n* = 13).
