# Supplementary material for: Multisensory Connections of Novel Linguistic Stimuli in Japanese as a Native Language and Referential Tastes
Source: Eur J Investig Health Psychol Educ. 2021 Sep 2;11(3):999–1010. doi: 10.3390/ejihpe11030074 (PMC8544189; doi:10.3390/ejihpe11030074)
Supplement: Supplementary file 1 [file ejihpe-11-00074-s001.zip › ejihpe-1335148-supplementary.pdf]

Supplemental Table S1. Japanese word stimuli (transliteration).

Word list 1	Word list 2	Word list 3	Word list 4
ヌヨ (nuyo)	ワユ (wayu)	ヌセ (nuse)	ヌニ (nuni)
ネケ (neke)	ヘオ (heo)	ムヌ (munu)	メフ (mefu)
ルエ (rue)	ルヨ (ruyo)	ロユ (royu)	ケネ (kene)
ツセ (tsuse)	テハ (teha)	ヌチ (nuchi)	ヌネ (nune)
ネユ (neyu)	ヘネ (hene)	ヘヨ (heyo)	ホヒ (hohi)
ムワ (muwa)	メヘ (mehe)	メワ (mewa)	ラヘ (rahe)
ラユ (rayu)	リワ (riwa)	ルモ (rumo)	ルラ (rura)
レヌ (renu)	レメ (reme)	ロワ (rowa)	エウ (eu)
ケメ (keme)	セヘ (sehe)	ソヌ (sonu)	テヨ (teyo)
ナネ (nane)	ヌオ (nuo)	ヌテ (nute)	ヌヤ (nuya)
ネセ (nese)	ノヌ (nonu)	ヘカ (heka)	ヘニ (heni)
ヘミ (hemi)	ホユ (hoyu)	ミメ (mime)	ムエ (mue)
ムユ (muyu)	メヌ (menu)	ユム (yumu)	ラヌ (ranu)
ラヨ (rayo)	ラレ (rare)	リヒ (rihi)	リヘ (rihe)
ルヘ (ruhe)	ルロ (ruro)	レソ (reso)	レロ (rero)
ロモ (romo)	ワソ (waso)	エオ (eo)	エヨ (eyo)
サウ (sau)	シヒ (shihi)	スヌ (sunu)	セア (sea)
セネ (sene)	ソミ (somi)	チオ (chio)	チニ (chini)
チメ (chime)	テヤ (teya)	ニネ (nine)	ニヨ (niyo)
ヌト (nuto)	ヌム (numu)	ネア (nea)	ネホ (neho)

These pseudowords associated with meaningless were selected from a previous psycholinguistic study of Japanese pseudowords [37]. The meaningfulness in the selected meaningless words ranged from 30 to 79 [37], while the non-association values in the selected meaningless words ranged from 35 to 85 [37].

Supplemental Table S2. Manufacturers and flavors of gustatory stimuli.

Stimulus	Manufacturer	Flavor
1	Ogontoh Co., Ltd.	kiwi
2	Asahi Group Foods, Ltd.	lemon
3	Meiji Sangyo Co., Ltd.	vinegar
4	Oshizaka Seika Co., Ltd.	Japanese bekko (pure caramel)
5	Meito Sangyo Co., Ltd.	white peach
6	Kanro Co., Ltd.	Japanese citrus
7	UHA Mikakuto Co., Ltd.	strawberry milk
8	NOBEL Confectionery Co., Ltd.	Lemon, including vitamin c
9	Meiji Co., Ltd.	dark chocolate
10	Sakumaseika Co., Ltd.	apple
11	Ribon Co., Ltd.	brown sugar, honey, and ginger
12	Pine Co., Ltd.	pineapple
13	NOBEL Confectionery Co., Ltd.	muscat including vitamin c
14	UHA Mikakuto Co., Ltd.	milk
15	Meiji Co., Ltd.	milk chocolate
16	Osakaya Seika Co., Ltd.	soy sauce radish
17	Asahi Group Foods, Ltd.	calpis
18	First Japan Co., Ltd.	butterscotch
19	Ribon Co., Ltd.	plum
20	Chikuho Seika Co., Ltd.	caramel

These gustatory stimuli were used in the evaluation phase and learning phase of G condition.

Supplemental Table S3. Results of correlation analyses (*n* = 13).

	SW	H	C	SO	AS	F	P	AR	D	SR	RT	SFR
SW	1											
H	-0.23**	1										
C	-0.13*	0.19**	1									
SO	-0.18**	0.10	0.56**	1								
AS	-0.34**	0.02	-0.11	0.02	1							
F	0.56**	-0.29**	0.07	-0.02	-0.39**	1						
P	0.60**	-0.25**	0.04	-0.02	-0.45**	0.81**	1					
AR	-0.35**	0.13*	0.27**	0.52**	0.32**	-0.33**	-0.34**	1				
D	0.56**	-0.29**	0.08	0.01	-0.45**	0.79**	0.85**	-0.32**	1			
SR	-0.07	-0.01	-0.04	-0.04	0.09	-0.10	-0.08	-0.09	-0.03	1		
RT	-0.01	-0.06	0.09	0.07	0.07	0.08	0.04	0.00	0.02	-0.08	1	
SFR	-0.14*	-0.02	0.03	-0.05	-0.02	-0.04	-0.09	0.00	-0.09	0.17**	-0.02	1

SW: sweetness; H: hardness; C: coolness; SO: sourness; AS: astringency; F: familiarity; P: preference; AR: arousal; D: deliciousness; SR: successful recognition; RT: response time of recognition memory phase; SFR: successful free recall.

** $p < 0.01$; * $p < 0.05$.
